# Measurement-Oriented Evaluation of Deep Learning Models for Automated Fetal Head Biometry in Prenatal Ultrasound

**DOI:** 10.3390/jcm15187127

**Published:** 2026-09-14

**Authors:** Hilal Gülsüm Turan Özsoy, Ebrar Elest Şen, Furkan Ertürk Urfalı, Gültekin Adanaş Aydın, Behiç Akyüz, Emre Dandıl

**Affiliations:** 1 Department of Radiology, Bursa City Hospital, 16250 Bursa, Türkiye; drfurkanurfali@gmail.com (F.E.U.); behicakyuz@gmail.com (B.A.); 2 Department of Computer Engineering, Faculty of Engineering, Bilecik Şeyh Edebali University, 11100 Bilecik, Türkiye; ebrarelestsen03@gmail.com; 3 Department of Obstetrics and Gynecology, Bursa City Hospital, 16250 Bursa, Türkiye; gadanas@gmail.com

**Keywords:** fetal ultrasound, fetal biometry, fetal head segmentation, prenatal assessment, automated biometric analysis, deep learning, U-Net, TransUNet, Residual U-Net

## Abstract

**Background/Objectives**: Accurate fetal head biometry, obtained via ultrasound imaging, is fundamental to prenatal assessment, as it enables fetal growth to be monitored and developmental abnormalities to be detected early. However, conventional manual measurements are time-consuming and operator-dependent, as well as being prone to inter-observer variability. This retrospective study proposes investigating whether conventional segmentation metrics accurately reflect downstream fetal biometric measurements. This would be achieved by evaluating three deep learning architectures on expert-selected, standard-plane fetal head ultrasound images. **Methods**: Three deep learning architectures, namely, U-Net, Residual U-Net and TransUNet were implemented and evaluated under identical conditions using a local clinical ultrasound dataset of 1918 images and masks from 206 pregnancies. Following segmentation, ellipse fitting was applied to both predicted and reference masks to derive head circumference (HC), biparietal diameter (BPD), and occipitofrontal diameter (OFD). Image-specific physical scaling was applied to convert pixel-based measurements into millimeters. The performance of models was assessed using conventional segmentation metrics, including the Dice Similarity Coefficient (DSC), the Intersection over Union (IoU), and the Hausdorff Distance (HD) metrics. This was complemented by a clinically meaningful biometric evaluation based on the mean absolute error (MAE) of the HC, BPD, and OFD measurements. **Results**: All three architectures achieved high segmentation performance, with DSC values above 0.98 and IoU values above 0.96. Residual U-Net produced the highest DSC and IoU values (0.9819 ± 0.0102 and 0.9647 ± 0.0196, respectively), whereas TransUNet produced the lowest HD value (1.8935 ± 0.8570 mm). For biometric measurements, the Residual U-Net produced the lowest MAE for HC (1.8877 ± 1.7689 mm) and OFD (0.8672 ± 0.8342 mm), while the TransUNet resulted in the lowest BPD MAE (0.7504 ± 0.7045 mm). Pairwise statistical analysis revealed significant differences in DSC between U-Net and Residual U-Net (*p* = 0.0172), as well as between Residual U-Net and TransUNet (*p* = 0.0119). However, no significant differences were observed between U-Net and TransUNet (*p* = 0.4726). For the biometric measurements, significant differences were only observed for the BPD MAE between the U-Net and TransUNet models (*p* = 0.0385), while the HC and OFD errors were statistically comparable across the model pairs. **Conclusions**: This study shows that high overlap-based segmentation performance does not necessarily lead to statistically significant or superior biometric measurement accuracy. The results emphasize the importance of using both conventional segmentation metrics and downstream, measurement-oriented evaluations when comparing deep learning models for fetal head biometry. However, as this study involved a retrospective technical evaluation of curated, expert-selected standard-plane images, the findings could not be interpreted as evidence of clinical effectiveness or immediate clinical applicability. Further validation is required using pregnancy-level separation, multiple observers, independent clinical measurements, pathological and technically challenging examinations, and multicenter external datasets.

## 1. Introduction

Fetal biometry is a fundamental component of obstetric practice. It is used to monitor fetal growth during pregnancy, estimate gestational age (GA) and detect potential developmental anomalies at an early stage [1,2,3]. Of these biometric parameters, head circumference (HC), biparietal diameter (BPD) and occipitofrontal diameter (OFD) are among the most widely used and decisive indicators in clinical decision-making processes due to their indirect relationship with brain development and fetal head volume [1,4]. An accurate assessment of these parameters is directly related to diagnosing fetal growth restriction, macrocephaly, microcephaly and other conditions that are associated with adverse perinatal outcomes [3].

Current international guidelines recommend performing fetal head biometry using standardized imaging planes and ellipse-based measurements to ensure consistency and reproducibility across examinations [5,6]. International guidelines (ISUOG) emphasize that HC measurement is a standard component of prenatal care, highlighting the critical impact of consistent measurements on the quality of clinical decision-making [7]. Specifically, HC and BPD measurements need to be taken from the transthalamic plane with a clear view of the cavum septi pellucidi and thalami, while making sure that the cerebellum and orbital structures are not visible [8]. Ellipse fitting around the outer skull contour is considered the preferred approach for HC estimation in routine clinical practice. Biometric measurements such as fetal HC are key inputs for regression-based models used to estimate GA. Even small changes in these measurements can affect the estimated GA and associated clinical decision-making processes [9,10].

Two-dimensional (2D) ultrasound is the most commonly used imaging method in fetal assessment due to its low cost, widespread availability and real-time imaging capabilities [11]. It also has the advantage of not involving ionizing radiation. However, a low signal-to-noise ratio, shadowing artefacts and operator-dependent variability in interpretation can complicate the diagnostic process. Despite these recommendations, ultrasound-based fetal biometry remains dependent on operator expertise. Acquiring appropriate scan planes, accurately delineating anatomical boundaries and placing measurement calipers correctly requires substantial training and experience. Consequently, manual measurements are often time-consuming and subject to intra- and inter-observer variability [12,13].

The growing use of artificial intelligence (AI) in medical imaging has opened up new possibilities for enhancing the efficiency and consistency of prenatal ultrasound examinations. Early studies primarily focused on the automatic detection of fetal standard planes, which are essential for reliable biometric assessment. Baumgartner et al. [12] proposed a convolutional neural network capable of identifying and localizing fetal standard planes in real time from freehand ultrasound acquisitions. The study demonstrated the feasibility of using deep learning to analyze obstetric ultrasounds. Further studies have built on these approaches to include automated anatomical segmentation and biometric estimation. van den Heuvel et al. [14] have developed a method that can automatically measure fetal HC from 2D ultrasound images. Acknowledging the significance of uncertainty quantification in clinical practice, Budd et al. [15] introduced a probabilistic deep learning framework that can provide confidence estimates alongside automated HC measurements. Their system generated real-time feedback on the reliability of predictions and showed mean HC deviations. Kim et al. [16] employed machine learning techniques to automatically measure fetal HC and BPD using ultrasound images. Zeng et al. [4] performed fetal head segmentation and automatic HC measurement in ultrasound images using deep learning models. Poojari et al. [17] investigated the accuracy of ultrasonographic fetal HC measurements and the factors that influence them. Slimani et al. [18] developed and prospectively validated deep learning models for the automated assessment of fetal biometry and amniotic fluid volume. They used a multicenter dataset comprising ultrasound images to do these assessments. Their findings showed that automated systems could perform well enough to be used in practice and highlighted the potential of AI. Similarly, Sundaramoorthy et al. [19] proposed a modular framework in which each biometric parameter-including HC-was optimized independently, in order to mimic the clinical workflow recommended by international guidelines. In addition, publicly available datasets for fetal biometric measurements and fetal head segmentation are limited. The release of the HC18 Grand Challenge dataset [14] was a significant development in this area, as it provided a publicly available benchmark for automatically measuring fetal HC from 2D ultrasound images.

Despite these encouraging developments, several important limitations remain unresolved. Firstly, the vast majority of studies continue to prioritize segmentation overlap metrics when reporting model performance. While these indices quantify the level of agreement between the predicted and reference masks at a pixel level, they do not necessarily reflect the accuracy of clinically relevant biometric measurements. Small deviations along critical portions of the skull contour may have minimal effect on these metrics, yet produce meaningful errors in HC, BPD or OFD estimation. Therefore, models with almost identical segmentation scores may differ substantially in their practical utility. Secondly, most published studies focus almost exclusively on HC estimation, whereas a comprehensive assessment of the fetal head in routine obstetric examinations also incorporates BPD and OFD measurements. These complementary measurements contribute to GA estimation, evaluation of cranial shape, and identification of pathological conditions such as microcephaly, macrocephaly, and dolichocephaly. Nevertheless, relatively few studies have simultaneously evaluated multiple biometric endpoints derived from a common segmentation framework. Thirdly, many existing algorithms have been developed and validated using public benchmark datasets acquired under relatively homogeneous imaging conditions. While these datasets are essential for methodological comparison, they may not adequately represent the diversity encountered in local clinical practice, including differences in ultrasound equipment, operator experience, patient populations and acquisition protocols. The external validity and clinical generalizability of automated fetal biometry systems therefore remain important concerns.

The current state of research shows significant progress in automated fetal head analysis but also highlights a significant discrepancy between excellent segmentation and clinical applicability. High overlap scores alone cannot guarantee accurate biometric measurements or provide meaningful support for obstetric decision-making. Therefore, evaluation frameworks that integrate conventional segmentation metrics with a direct assessment of biometric measurement errors are needed to better characterize the clinical utility of deep learning models. The objective of this study is not just to automate conventional fetal head measurements, but also to investigate whether the accuracy of biometric measurements is adequately reflected by conventional image-overlap metrics used to assess segmentation performance. Therefore, this study primarily addresses this gap by investigating whether superior segmentation performance translates into improved HC, BPD and OFD estimation using a local clinical fetal ultrasound dataset acquired under routine practice conditions. The main contributions of this study can be summarized as follows:

i. A measurement-oriented deep learning framework was developed and evaluated for fetal head segmentation and automated extraction of clinically relevant biometric measurements, including HC, BPD, and OFD, from retrospectively selected standard-plane fetal ultrasound images.
ii. Three widely used segmentation architectures, namely U-Net, Residual U-Net, and TransUNet, were systematically implemented and evaluated under consistent experimental conditions using the same dataset partitions and evaluation protocol.
iii. A measurement-oriented evaluation strategy was adopted in addition to conventional segmentation metrics. The study assessed whether differences in segmentation quality were reflected in the accuracy of HC, BPD, and OFD measurements derived from the predicted fetal head contours.
iv. The relationship between segmentation performance and downstream biometric accuracy was investigated using descriptive and pairwise statistical analyses. The results demonstrate that higher overlap-based segmentation performance does not necessarily result in more accurate biometric measurements, highlighting the importance of evaluating contour-derived measurements alongside conventional segmentation metrics.
v. A dataset of 1918 clinically acquired fetal ultrasound images from 206 pregnancies was used. Images suitable for fetal head biometric assessment were selected and reviewed by experts. The study also incorporated image-specific physical scaling and reproducibility-oriented implementation details, as well as data augmentation analysis and qualitative Grad-CAM visualization, to provide a more comprehensive evaluation of the proposed measurement-oriented framework.

## 2. Materials and Methods

**Figure 1:**
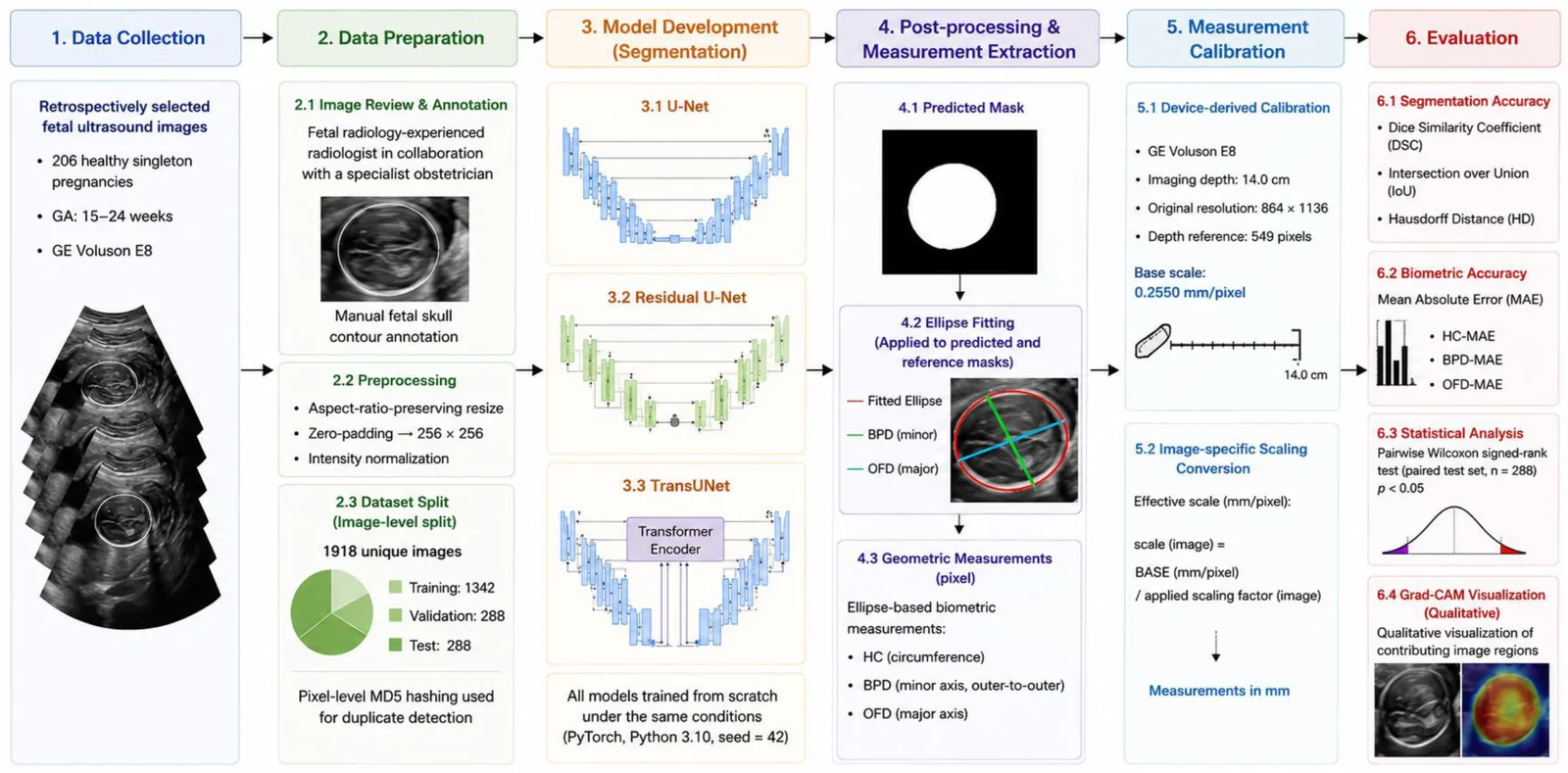
Overview of the proposed measurement-oriented framework for automated fetal head biometry from prenatal ultrasound images.

The overall workflow of the proposed framework is shown in Figure 1. The workflow consists of six main stages, including data collection, data preparation, model development, post-processing, measurement extraction, measurement calibration and evaluation. Prenatal fetal ultrasound images were annotated to generate fetal head masks and prepared for model development using defined pre-processing and dataset partitioning procedures. Prenatal ultrasound images are first annotated to generate fetal head masks, and are then preprocessed by resizing and normalizing the intensity. Three segmentation architectures, namely U-Net, Residual U-Net, and TransUNet, were trained and evaluated for fetal head segmentation. The predicted masks were then processed using ellipse fitting to extract biometric measurements of the fetal head, including HC, BPD and OFD. The extracted measurements were then calibrated from pixels to millimeters using the image-specific scaling procedure. Finally, the performance of the model was evaluated using metrics for segmentation and biometric accuracy, as well as through pairwise comparative statistical analyses and Grad-CAM-based qualitative visualization. This workflow allows both the performance of the segmentation and the accuracy of the clinically relevant fetal biometric measurements derived from the predicted fetal head contours to be assessed.

### 2.1. Dataset

**Figure 2:**
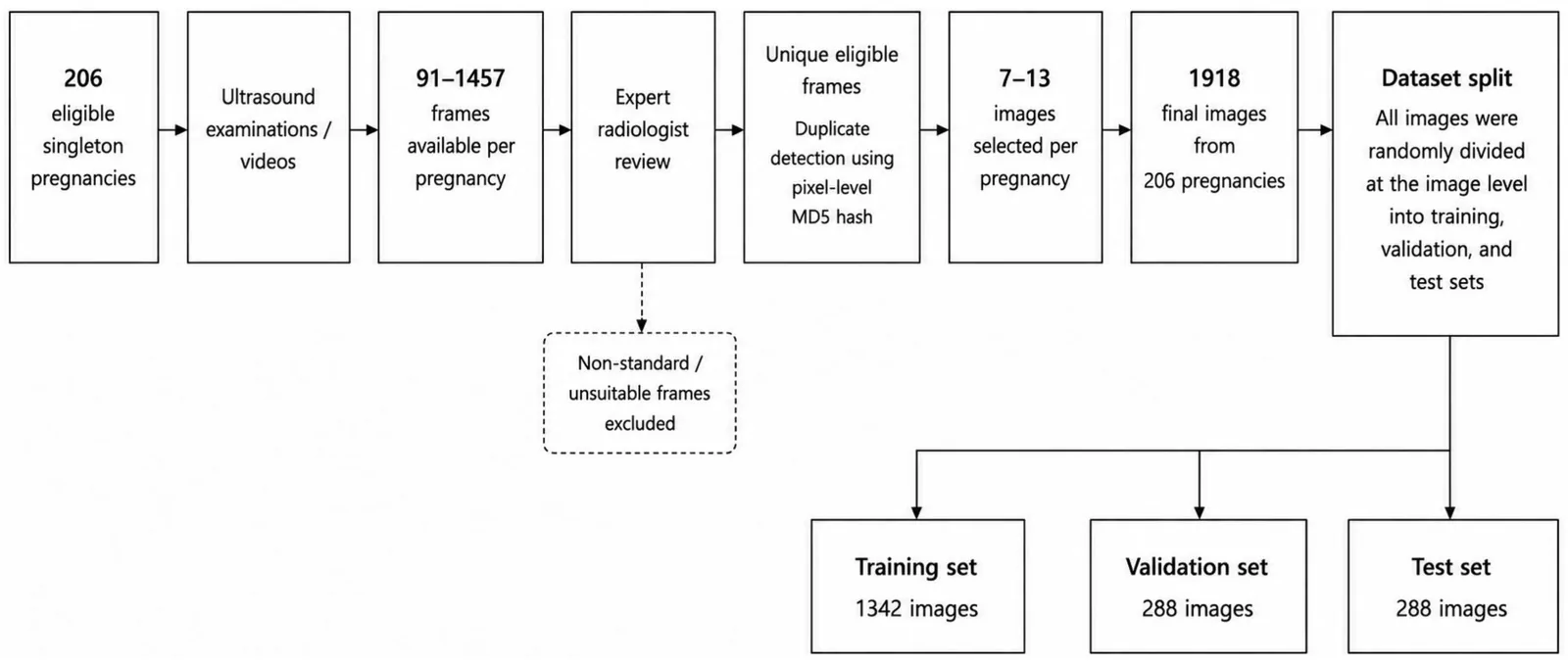
Workflow for selecting patients and images and splitting image-level datasets.

The development of original datasets is a critical step in designing robust deep learning models for medical image analysis. While several studies have investigated fetal head segmentation and biometric estimation using ultrasound datasets, datasets derived from local clinical practice remain limited. To address this, a study-specific ultrasound dataset of fetal heads was created to enable the automatic segmentation of fetal heads and the extraction of relevant clinical biometric measurements. The dataset was retrospectively constructed using prenatal ultrasound examinations performed at Bursa City Hospital, Bursa, Türkiye. Ultrasound scans were obtained from 206 healthy singleton pregnancies involving women aged between 18 and 40 years. The mean maternal age was 25.95 ± 4.97 years with a median of 25.50 years (interquartile range (IQR): 22.00–30.00). The GA of the fetuses ranged from 15 to 24 weeks, and all examinations were conducted between July 2025 and January 2026 as part of routine obstetric assessments. Between 91 and 1457 frames were initially available from each pregnancy. No fixed temporal sampling interval was applied. Instead, an experienced radiologist reviewed the candidate frames and selected those demonstrating an appropriate standard plane for fetal head biometry. To avoid redundant observations, 7 to 13 unique frames were selected from each pregnancy. To reliably identify unique images, pixel-level MD5 hash comparison was used instead of filename-based matching. This approach enabled the detection of images with identical pixel content even when they had different file names. During the expert selection of 7–13 unique standard-plane images per pregnancy, non-identical but visually redundant frames were also minimized. The gestational weeks and obstetric ultrasound findings for the patients in the dataset were carefully documented. Figure 2 shows the workflow for selecting patients and images used in the study. This generated 1342 images for training, 288 for validation and 288 for testing.

All ultrasound examinations were performed using a General Electric (GE) Voluson E8 (GE Healthcare Austria GmbH & Co OG, Zipf, Austria) ultrasound system with a C2-9 MHz convex transducer in the Radiology Department at Bursa City Hospital. Rather than collecting still images directly, prenatal ultrasound videos recorded during routine clinical examinations were utilized. Individual image frames were extracted from the recorded videos for subsequent expert review. All available extracted frames were visually reviewed by a single radiologist with experience in fetal radiology, working in collaboration with a specialist obstetrician. Only images that were suitable for fetal head biometric assessment and that represented the standard transthalamic plane were selected for further analysis. These images were required to show an appropriately symmetrical fetal cranial contour and the relevant anatomical landmarks, including the bilateral thalami and, when identifiable, the midline cavum septi pellucidi, with no visualization of the cerebellum within the measurement plane. Images with incomplete visualization of the fetal calvarium, severe acoustic shadowing, motion artefacts or poor image quality were excluded. The outer contour of the fetal skull was manually delineated at pixel level for each selected image to generate the reference segmentation masks. The annotation process was performed under the supervision of an experienced obstetrician with expertise in fetal ultrasound imaging to ensure anatomical consistency and annotation accuracy. After annotation, the final dataset comprised 1918 image–mask pairs representing clinically relevant fetal head measurements.

All ultrasound images were anonymized to remove patient-identifiable information prior to data processing. The study was conducted in accordance with the ethical principles outlined in the Declaration of Helsinki, complying with the regulations of the Turkish Personal Data Protection Law (KVKK). Ethical approval was obtained from the Scientific Research Ethics Committee of Bursa City Hospital (Approval No. 2026-06/3; Approval Date: 25 March 2026). Written informed consent was obtained from all pregnant women participating in the study before their inclusion.

Using a locally acquired clinical dataset that encompasses real-world variability in image acquisition conditions provides a realistic framework for evaluating segmentation performance and the reliability of automated fetal biometric measurements in routine obstetric practice. The main characteristics of the local fetal ultrasound dataset used in this study are summarized in Table 1, including demographic information, imaging conditions, annotation details, calibration parameters, and dataset partitioning employed for model development and evaluation.

**Figure 3:**
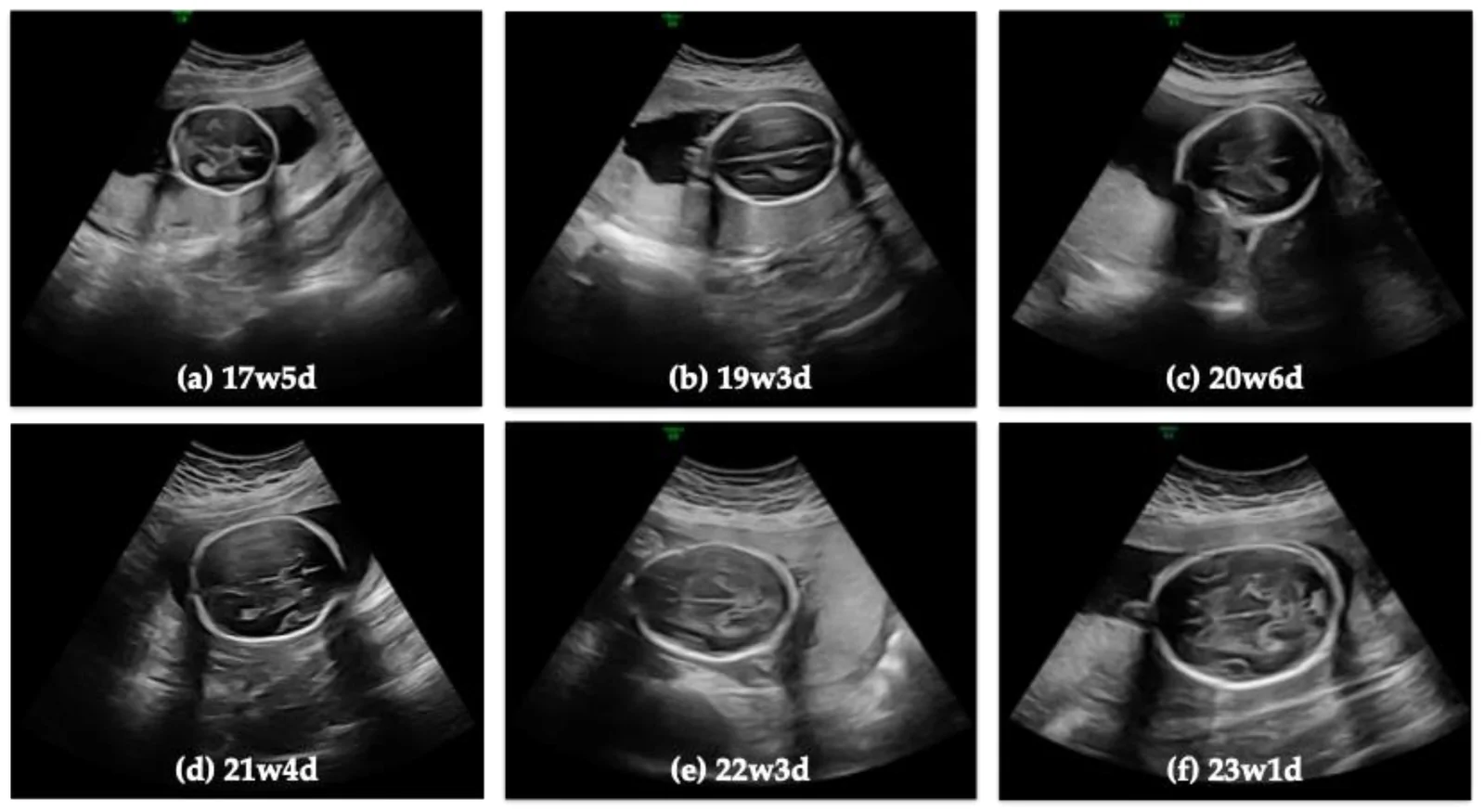
Examples of standard fetal head ultrasound images from the locally acquired clinical dataset across different GAs. Examples obtained from six different pregnancies are shown, corresponding to gestational ages of (**a**) 17w5d, (**b**) 19w3d, (**c**) 20w6d, (**d**) 21w4d, (**e**) 22w3d, and (**f**) 23w1d. All images represent standard transthalamic planes used for routine fetal head biometry and demonstrate the variability in fetal anatomy and ultrasound image characteristics across gestation.

To ensure consistent assessment of the fetal head, the study only included singleton pregnancies in the second trimester. Only images that were suitable for fetal head biometric assessment and that represented the standard transthalamic plane were included. These images demonstrated an appropriately symmetrical fetal cranial contour and the relevant anatomical landmarks for identification of the measurement plane. This included the bilateral thalami and, when identifiable, the midline cavum septi pellucidi, with no cerebellum within the measurement plane. Figure 3 presents representative examples from six different pregnancies. These images correspond to GA ranging from 17 weeks and 5 days to 23 weeks and 1 day and demonstrate the variation in fetal head size and ultrasound appearance across the studied GA range. These expert-selected, standard-plane images were subsequently used for manual skull contour annotation, segmentation analysis and ellipse-based derivation of HC, BPD and OFD.

The reference standard for fetal head biometry was constructed directly from manually annotated ground-truth segmentation masks. The reference HC, BPD and OFD values were neither obtained from clinical reports nor extracted from measurement overlays displayed on ultrasound images. Instead, the biometric measurements were calculated from the corresponding ground-truth masks using the same ellipse-fitting procedure that was applied to the predicted segmentation masks. This ensured that the reference and predicted biometric measurements were obtained using an identical computational procedure. The manually annotated mask represented the fetal skull contour. An ellipse was fitted to this contour and the geometry of the ellipse was subsequently used to derive the fetal head biometric parameters. HC was calculated from the fitted ellipse representing the outer skull contour. BPD was determined using the outer-to-outer convention, while OFD was derived from the orthogonal cranial dimension of the fitted ellipse.

### 2.2. Image Pre-Processing

In this study, fetal ultrasound images were processed using a standard pre-processing pipeline before being supplied to the deep learning models. The original ultrasound images had a fixed resolution of 864 × 1136 pixels, were converted to grayscale, and were then resized to a square input. To avoid geometric distortion when resizing rectangular images directly to a square input, a resizing strategy that preserves the aspect ratio with zero-padding was adopted. First, the longer image dimension was scaled to 256 pixels, then, the shorter dimension was completed to 256 pixels using zero-padding. Thus, the original aspect ratio of the ultrasound images was preserved during pre-processing and a resolution of 256 × 256 pixels was used for all three model architectures (U-Net, Residual U-Net and TransUNet). The scaling factor applied during this resizing operation was recorded separately for each image.

A calibration procedure was established to convert pixel-based biometric measurements into physical units, based on the depth scale displayed by the ultrasound system. All images were acquired using the same ultrasound device (a GE Voluson E8) at a fixed imaging depth. This calibration was established after confirming that the same imaging device, depth setting and original image resolution were used throughout the dataset. As the images were resized prior to model inference, the effective physical pixel spacing was adjusted according to the recorded scaling factor for each image. Specifically, the effective pixel-to-millimeter conversion factor was calculated by dividing the base calibration factor by the applied image scaling factor. Consequently, biometric measurements were converted from pixels to millimeters using an image-specific physical scale rather than applying a single, fixed pixel-to-millimeter conversion factor to the resized images. This procedure accounts for the resizing operation while preserving the physical dimensions represented in the original ultrasound images.

To ensure stable model training, the images were normalized to the range [0, 1]. No speckle-reduction filtering or other image enhancement pre-processing was applied in order to preserve the original boundary and texture information present in the ultrasound images. To ensure compatibility with the TransUNet architecture, the images were converted from grayscale to three-channel RGB format.

The segmentation masks were subjected to morphological filling operations to eliminate any contour discontinuities that might have arisen during manual annotation, with the intention of improving the model’s edge detection performance. The masks were then converted to binary format.

### 2.3. Calibration and Scaling

A calibration procedure was established to convert pixel-based measurements into physical units using the depth scale displayed by the clinical ultrasound system (GE Voluson E8). All ultrasound images in the dataset were acquired using this system with a fixed imaging depth of 14.0 cm and an original resolution of 864 × 1136 pixels. The depth scale displayed on the left of the ultrasound image was measured in ImageJ software v1.54g [20] using the straight-line measurement tool. The distance corresponding to the full 0–14.0 cm depth range was measured as 549 pixels. The base pixel-to-millimeter conversion factor was therefore calculated as 140 mm/549 pixels, or 0.2550 mm/pixel. This value was used as the reference physical scale for the subsequent image-specific scaling procedure.

The measurements obtained from the ultrasound images were initially calculated in pixels. To convert these pixel-based measurements into physical units, an image-specific calibration and scaling procedure was applied. Because the original ultrasound images were resized for model input while preserving their aspect ratio, the effective physical pixel spacing was adjusted according to the resizing factor applied to each image. The effective pixel-to-millimeter conversion factor $s_{mm/pixel}^{\left(i\right)}$ was calculated using the base calibration factor $S_{base}$ and the image-specific resizing factor $s_{i}$, as defined in Equation (1). The final biometric measurements in millimeters were then obtained by multiplying the pixel-based measurements by the corresponding image-specific physical pixel spacing, as shown in Equation (2). Here, $S_{base}$ represents the base calibration factor of 0.2550 mm/pixel derived from the depth scale displayed by the ultrasound system, whereas $s_{i}$ represents the resizing factor applied to the i-*th* image during aspect-ratio-preserving preprocessing. $s_{mm/pixel}^{\left(i\right)}$ therefore represents the effective physical pixel spacing for the corresponding image. $M_{pixel}^{\left(i\right)}$ denotes the biometric measurement obtained from the i-th image in pixels, and $M_{mm}^{\left(i\right)}$ represents the corresponding measurement in millimeters. This image-specific scaling procedure accounts for the effect of resizing while preserving the original aspect ratio of the ultrasound images.

(1) $$s_{mm/pixel}^{\left(i\right)}=\frac{S_{base}}{s_{i}}$$

(2) $$M_{mm}^{\left(i\right)}=M_{pixel}^{\left(i\right)}\;\times \;s_{mm/pixel}^{\left(i\right)}$$

This calibration and scaling procedure was applied after the model was predicted, in order to convert the pixel-based biometric measurements into millimeters. As the aspect ratio of the original ultrasound images was preserved during pre-processing, no independent horizontal or vertical geometric correction was necessary. Using an image-specific scaling factor ensured that the physical dimensions of the predicted fetal head contours were calculated according to the original image scale, rather than the 256 × 256 input dimensions of the model. This approach minimized potential systematic measurement errors resulting from image resizing and provided a consistent physical scale for estimating HC, BPD and OFD.

### 2.4. Deep Learning Architectures

In recent years, deep learning-based methods have played a transformative role in medical image segmentation. The U-Net architecture, in particular, has become the widely accepted solution for medical segmentation tasks thanks to its symmetric encoder–decoder structure and skip connections [21]. Self-configuring frameworks such as nnU-Net have demonstrated the ability to automatically adapt their preprocessing, network configuration, training, and post-processing strategies to different datasets [22]. However, CNNs largely focus on local features, so they may exhibit performance limitations when dealing with weak edges and contour discontinuities, which are frequently observed in ultrasound images [23]. To address these limitations, transformer-based architectures [24] and hybrid approaches that can model global context using self-attention mechanisms such as TransUNet have been developed [25,26]. Hybrid transformer-CNN models have been reported to produce promising results in challenging situations where the anatomical form needs to be preserved holistically.

To investigate the impact of different architectural designs on fetal head segmentation and subsequent biometric estimation, three representative deep learning models such as U-Net, Residual U-Net and TransUNet were evaluated in this study. These architectures were selected because they represent three distinct paradigms in medical image segmentation, namely conventional encoder–decoder convolutional networks, residual learning-based convolutional networks, and hybrid convolutional-transformer architectures.

#### 2.4.1. U-Net

The U-Net architecture is one of the most widely adopted for biomedical image segmentation. Originally proposed by Ronneberger et al. [27], this encoder–decoder network is designed to capture contextual information while preserving localization accuracy. The encoder path progressively reduces the spatial dimensions of the input image by repeatedly applying convolutional and pooling operations, thereby learning increasingly abstract feature representations. Conversely, the decoder path restores spatial resolution by performing successive upsampling operations [28].

A key feature of the U-Net architecture is the incorporation of skip connections between the corresponding encoder and decoder stages [29]. These connections enable the high-resolution feature maps extracted during the encoding process to be combined directly with the decoder representations [30]. This facilitates the recovery of fine anatomical details, which are essential for accurate segmentation. Due to its simplicity, computational efficiency and robust performance in settings with limited data, U-Net has become the benchmark architecture for many medical imaging applications, including the analysis of fetal ultrasounds. In the context of fetal head segmentation in particular, U-Net’s ability to integrate global contextual information with precise boundary localization makes it well-suited to delineating the fetal skull contour, even in the presence of noise and heterogeneous image appearances.

#### 2.4.2. Residual U-Net

Although the U-Net model demonstrates strong segmentation performance, increasing the depth of the network can introduce optimization challenges, such as vanishing gradients and a decline in training accuracy [31,32]. To overcome these limitations, the Residual U-Net architecture was developed by incorporating residual learning into the U-Net framework. Residual learning was first introduced by He et al. [33] in the form of the ResNet architecture. Rather than learning the desired mapping directly, residual blocks estimate residual functions with respect to the input features. This strategy uses identity shortcut connections to facilitate gradient propagation and enable the effective training of deeper networks [34,35].

The Residual U-Net replaces the conventional convolutional blocks of the U-Net with residual units, while maintaining the encoder–decoder topology and skip connections [36]. Combining multiscale feature extraction with residual learning enhances feature reuse, improves optimization stability and increases robustness against noise and image variability. Given the challenging characteristics of ultrasound imaging, including low contrast, acoustic artifacts, and indistinct tissue boundaries, Residual U-Net has the potential to improve contour delineation and preserve the geometric characteristics required for reliable fetal biometric measurements.

**Figure 4:**
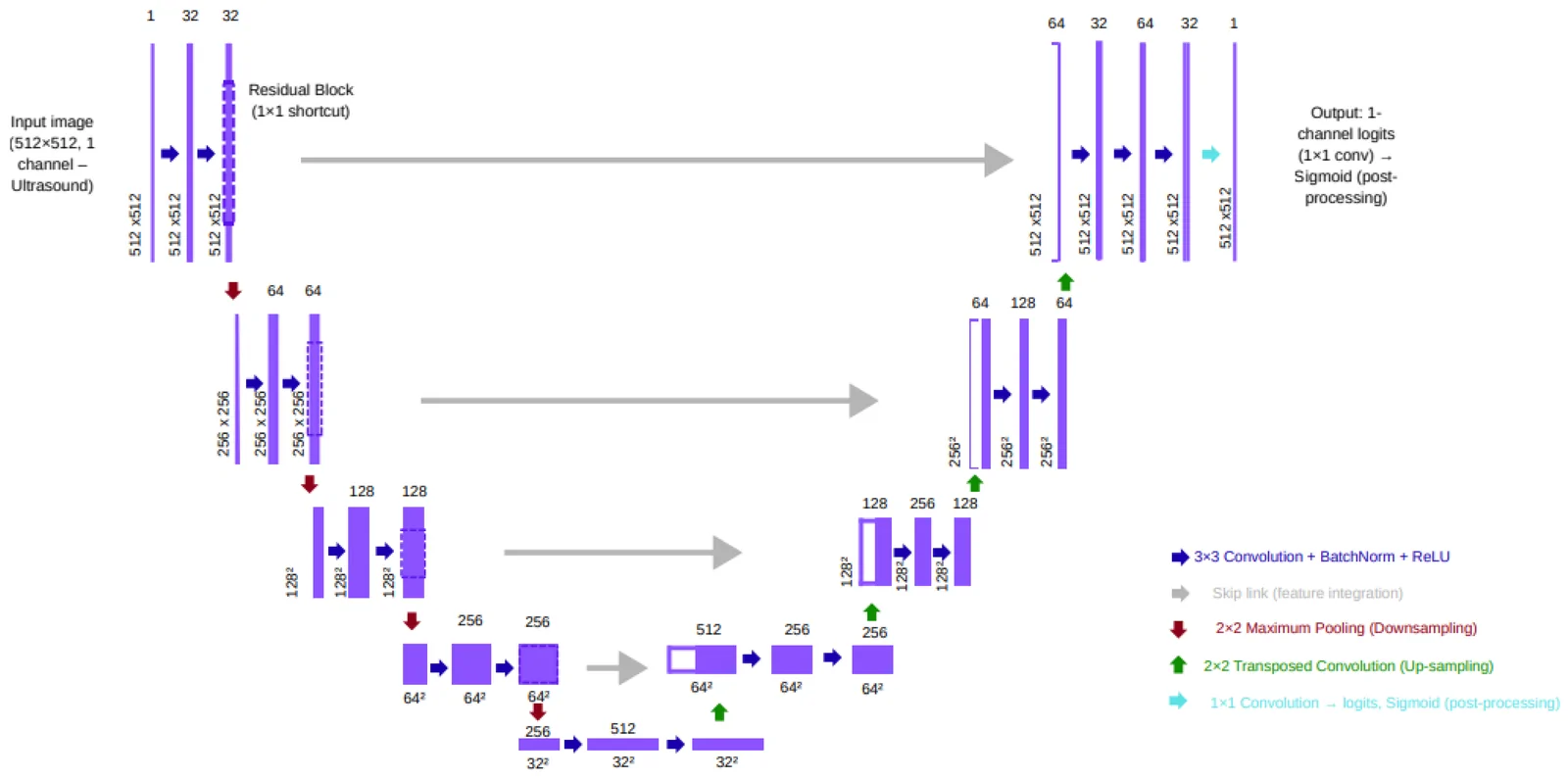
Detailed architecture of the proposed Residual U-Net segmentation network.

The detailed architecture of the Residual U-Net model employed in this study is illustrated in Figure 4. The network has an encoder–decoder structure comprising residual blocks, max-pooling operations, transposed convolutions and skip connections. The encoder progressively extracts hierarchical feature representations while reducing the spatial resolution of the input ultrasound image. At the bottleneck layer, high-level semantic features are learned before being transferred to the decoder. The decoder then reconstructs the segmentation mask by performing successive upsampling operations and fusing features with the corresponding encoder layers via skip connections. Residual blocks facilitate gradient propagation and improve feature learning, enabling the fetal head contours in ultrasound images to be delineated more accurately.

#### 2.4.3. TransUNet

Recent advances have demonstrated the effectiveness of transformer architectures in capturing long-range dependencies and modelling global contextual relationships [37]. Unlike convolutional operations, which focus on local neighborhoods, transformers use self-attention mechanisms to establish interactions between distant regions of an image. TransUNet, proposed by Chen et al. [26], combines the advantages of convolutional neural networks and vision transformers within a unified segmentation framework. In this architecture, convolutional layers first extract low-level spatial representations from the input image. These feature maps are then transformed into token embeddings and processed by Transformer encoder layers to model global contextual information [38]. The resulting representations are then decoded using a U-Net-like decoder with skip connections to produce high-resolution segmentation outputs.

The hybrid nature of TransUNet allows local texture information and global semantic relationships to be exploited simultaneously. This is particularly advantageous for fetal ultrasound images, where the fetal skull contour may be obscured by noise, attenuation artifacts or incomplete boundaries. Leveraging self-attention mechanisms enables TransUNet to enhance shape consistency and improve the delineation of anatomically relevant structures.

**Figure 5:**
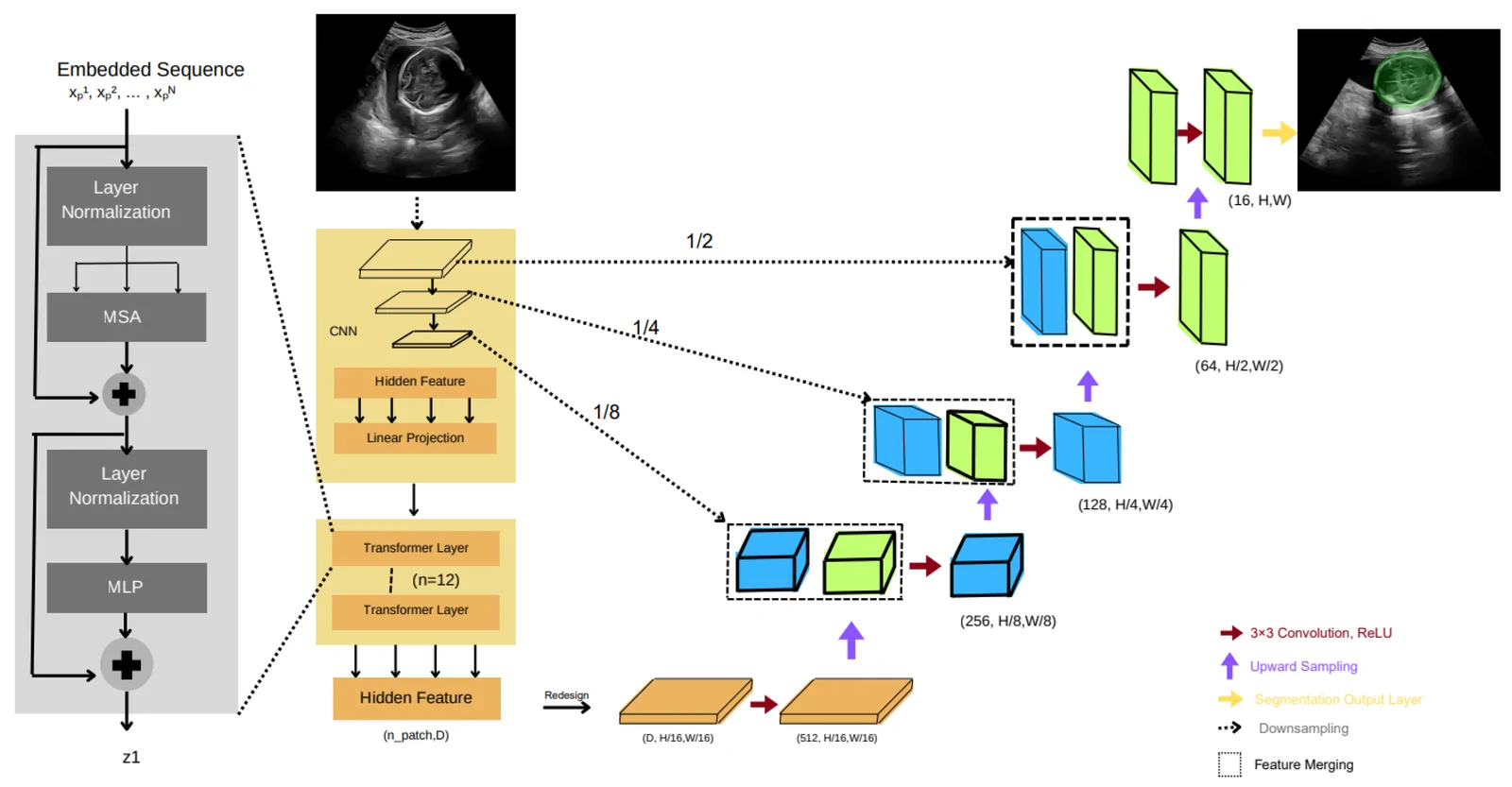
The architecture of the TransUNet framework integrating CNN and Transformer components used for fetal head segmentation.

The detailed architecture of the TransUNet model is presented in Figure 5. The model incorporates both CNN and transformer encoders within a unified encoder–decoder framework. Initially, low-level spatial features are extracted from the input ultrasound image using convolutional layers. These feature maps are then transformed into token embeddings and processed by Transformer encoder blocks, which consist of multi-head self-attention (MSA) and multilayer perceptron (MLP) layers. This enables the modelling of long-range spatial dependencies and global contextual information. These encoded representations are then passed to a U-Net-like decoder where transposed convolutions and skip connections progressively restore spatial resolution. Feature fusion between the encoder and decoder stages enables the recovery of fine anatomical details, resulting in the accurate delineation of fetal head contours and improved segmentation performance.

### 2.5. Hyperparameter Optimization and Implementation Details

All models were implemented using the PyTorch library with Python 3.10. Each architecture was trained using the optimization method best suited to its learning dynamics. To ensure a fair comparison, the models were trained using the same training, validation and the held-out test sets. All models were trained from scratch and none of the evaluated architectures used pretrained weights. All input images were resized to 256 × 256 pixels prior to being supplied to the networks. No patch extraction was performed. No connected-component filtering or component selection procedure was applied to the predicted masks. The binary predictions were used directly for subsequent evaluation and extraction of biometric measurements. No additional post-processing was applied to the segmentation masks themselves. For the biometric analysis, the predicted fetal head contours were subjected to ellipse fitting in order to derive the HC, BPD and OFD measurements.

The Adam optimization method was used for U-Net and Residual U-Net with an initial learning rate of 1 × 10^−4^ and a ReduceLROnPlateau scheduler (factor 0.5, patience 5), tracking validation Dice. TransUNet was trained using stochastic gradient descent (SGD) with momentum of 0.9, weight decay of 1 × 10^−4^, an initial learning rate of 1 × 10^−2^, and a polynomial learning rate reduction strategy. A weighted combination of the Dice and binary cross-entropy (BCE) terms was used as the loss function, as seen in Equation (3).

(3) $$Loss=0.5\;\times \;L_{BCE}+1.5\;\times \;L_{Dice}$$

The weighting in Equation (3) penalizes both regional overlap errors and pixel-level misclassifications. It is therefore well-suited to the class imbalance (background and head) that is typically observed in ultrasound segmentation. All training runs used 50 epochs, gradient clipping (max norm = 1.0), automatic mixed-precision (AMP) training to improve memory efficiency and a fixed random seed. Model selection was performed based on the epoch with the highest validation Dice score, with the test subset used solely for final evaluation. All experiments were conducted on workstations equipped with an NVIDIA GeForce GTX 1080 graphics card with 8 GB VRAM. The basic training configurations are summarized in Table 2. The number of trainable parameters was recorded for each architecture, providing a quantitative indication of model complexity. The U-Net contained 7.765.409, the Residual U-Net contained 8.115.073 and the TransUNet contained 105.322.001. Therefore, TransUNet had substantially higher parameter complexity than the two CNN-based architectures. During the preparation of this manuscript, the authors used ChatGPT (GPT-5.3) solely for the purposes of improving the language, grammar, and readability of the manuscript. The authors have reviewed and edited the output and take full responsibility for the content of this publication.

## 3. Results

The performance of the proposed deep learning-based segmentation models on the dataset was analyzed comparatively in the experimental analyses. The approach developed in this study considers not only the models’ pixel-level performance, but also the accuracy of biometric measurements and visual explainability, both of which are critical for clinical diagnosis. The experimental findings are presented alongside quantitative performance metrics, an overview of the training process and visual activation maps.

**Figure 6:**
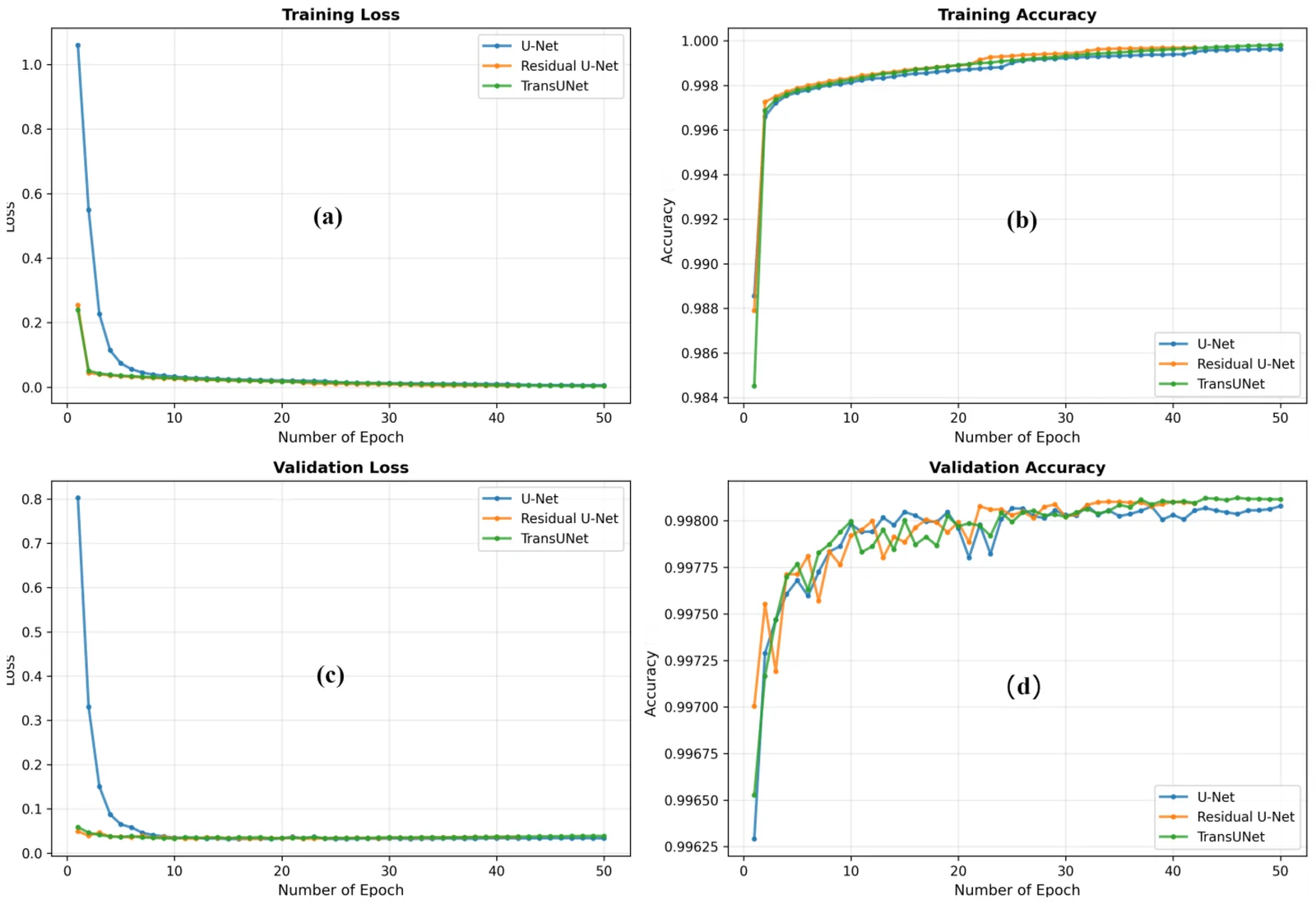
Training and validation performance curves of the evaluated deep learning models such as U-Net, Residual U-Net, and TransUNet. (**a**) Training loss, (**b**) training accuracy, (**c**) validation loss, and (**d**) validation accuracy over 50 training epochs.

To enable fair comparison of the evaluated architectures, all models were trained and tested under identical experimental conditions. The dataset was randomly split into image-level subsets, with 70% for training (N = 1342), 15% for validation (N = 288), and 15% for testing (N = 288) using a fixed random seed (seed = 42). This ensured reproducibility and minimized potential sampling bias during model evaluation. Figure 6 shows the training and validation curves of the three evaluated segmentation models. As can be seen in Figure 6a,c, all models rapidly converged during the initial training epochs, with substantial decreases in both training and validation losses before stable values were reached. Similarly, the training and validation accuracies steadily increased throughout the optimization process shown in Figure 6b,d, ultimately succeeding for all models. The close agreement between the training and validation curves indicates that the adopted training strategy successfully minimized overfitting whilst maintaining strong generalization capability across the evaluated architectures.

We quantitatively evaluated the segmentation performance of the proposed models using the Dice Similarity Coefficient (DSC), the Intersection over Union (IoU) metric, and the Hausdorff Distance (HD) metric. These metrics assess the overlap and boundary agreement between the predicted segmentation mask and the corresponding ground-truth annotation. DSC measures the overlap between predicted (P) and reference (G) segmentation masks, as defined in Equation (4).

(4) $$DSC=\frac{2\left|P\cap G\right|}{\left|P\right|+\left|G\right|}$$

The IoU metric is used to measure the ratio between the intersection and the union of the predicted and reference masks, as shown in Equation (5). While DSC and IoU evaluate regional overlap, HD was used to quantify the maximum boundary discrepancy between the predicted and reference fetal head contours. For each image, the symmetric maximum Hausdorff distance was calculated as the maximum of the two directed Hausdorff distances between *P* and *G*. It is defined in Equations (6) and (7).

(5) $$IoU=\frac{\left|P\cap G\right|}{\left|P\cup G\right|}$$

(6) $$hd(P,G)=\underset{p\in P}{\sup}\;\underset{g\in G}{\inf}\;d\left(p,g\right)$$

(7) $$HD(P,G)=max\left(hd(P,G),\;hd(G,P)\right)$$

where $d\left(\cdot \;,\;\cdot \right)$ denotes the Euclidean distance between two boundary points. Thus, the HD used in this study is the maximum Hausdorff distance. A smaller HD value indicates closer agreement between the predicted and reference boundaries.

Following segmentation, biometric measurements were extracted from the predicted fetal head masks by fitting an ellipse to the outer contour of the skull. The ellipse parameters were obtained by applying the least-squares ellipse fitting algorithm to the outer boundary of the predicted segmentation mask. This approach is consistent with the ellipse-based measurement methodology that is routinely implemented in clinical obstetric ultrasound systems. The fitted ellipse was characterized by its semi-major axis (a) and semi-minor axis (b), and the HC was estimated using Ramanujan’s approximation for the circumference of an ellipse in Equation 8. The Ramanujan approximation was used for the HC ellipse perimeter. All measurements calculated in pixels were converted to millimeters using the calibrated effective pixel size.

(8) $${HC}_{px}\approx \pi \left[3\left(a+b\right)-\sqrt{\left(3a+b\right)\left(a+3b\right)}\right]$$

where ${HC}_{px}$ denotes the estimated head circumference in pixels. The BPD and the OFD were calculated directly from the fitted ellipse as the lengths of the minor and major axes, respectively, using Equations (9) and (10).

(9) $${BPD}_{px}=2b$$

(10) $${OFD}_{px}=2a$$

To obtain clinically meaningful measurements, all pixel-based biometric values were converted into millimeters using the calibration factor specific to the dataset, which was determined from the ultrasound system. The final HC, BPD and OFD measurements were therefore reported in millimeters and compared with the corresponding reference measurements derived from the ground-truth masks. The level of agreement between the automatically estimated biometric measurements and the corresponding reference measurements was quantified using the mean absolute error (MAE). Let $M_{i}^{P}$ and $M_{i}^{G}$ denote the predicted and reference biometric measurements HC, BPD and OFD for the i-*th* image, respectively. The MAE is computed using Equation (11).

(11) $$MAE=\frac{1}{N}\;\sum_{i=1}^{N}\left|M_{i}^{P}-M_{i}^{G}\right|$$

where *N* represents the total number of test images. Lower MAE values indicate greater consistency between automatically and manually estimated biometric parameters, reflecting superior clinical measurement accuracy.

**Figure 7:**
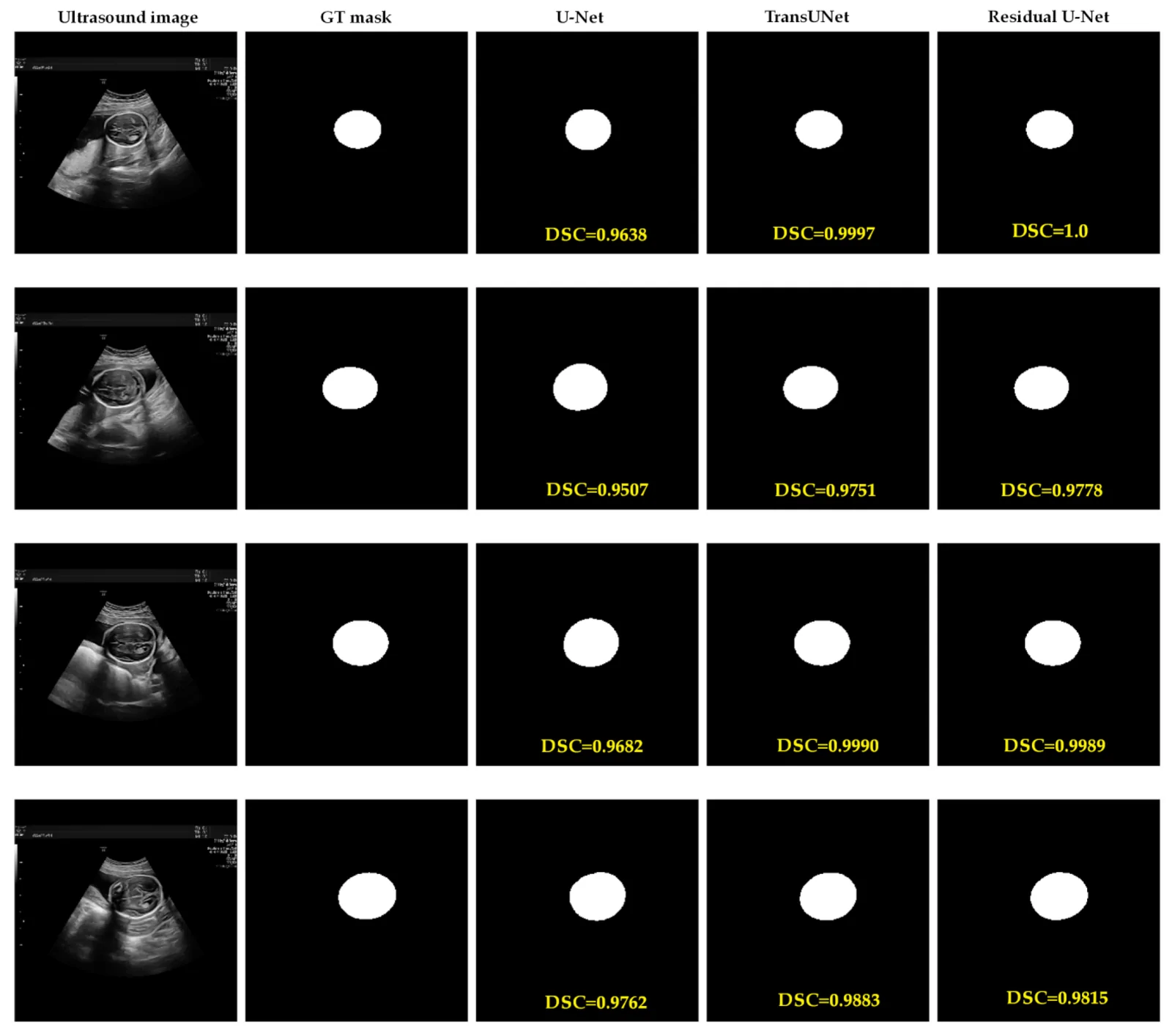
Qualitative comparison of fetal head segmentation results obtained by the evaluated deep learning models.

Figure 7 provides examples of the segmentation results obtained using U-Net, TransUNet and Residual U-Net, alongside the corresponding ground truth masks. Overall, all three architectures successfully delineated the fetal head region, producing segmentation masks that closely corresponded to the reference annotations. The high DSC values observed in the representative cases demonstrate the models’ performance across different ultrasound appearances and fetal head configurations. Of the evaluated architectures, Residual U-Net generally produced the most consistent and anatomically coherent contours, with smooth boundaries and the closest agreement with the ground-truth masks. This qualitative observation is consistent with the quantitative results, in which Residual U-Net achieved the highest DSC value of the three models. U-Net and TransUNet likewise generated visually accurate masks, although minor differences in contour shape and boundary localization were apparent across cases. The close visual similarity of the predicted masks illustrates that the differences between the models are relatively subtle at the pixel-overlap level. These findings demonstrate that all three architectures are capable of reliable fetal head segmentation. However, the slightly more consistent contour representation obtained with Residual U-Net may be advantageous for the subsequent extraction of geometric biometric measurements. Overall, the qualitative results support the quantitative segmentation findings and motivate further evaluation of biometric accuracy in addition to conventional segmentation metrics.

Table 3 summarizes the quantitative segmentation performance of the three deep learning architectures on the test set. Model performance was evaluated using DSC, IoU and HD. Higher DSC and IoU values indicate greater overlap between the predicted and reference masks, while lower HD values indicate more accurate boundary localization. All three architectures demonstrated high segmentation performance, with DSC values ranging from 0.9812 to 0.9819, and IoU values ranging from 0.9633 to 0.9647. Residual U-Net achieved the highest DSC and IoU values (0.9819 ± 0.0102 and 0.9647 ± 0.0196, respectively), followed by TransUNet with values of 0.9815 ± 0.0101 and 0.9638 ± 0.0090, respectively. U-Net yielded DSC and IoU values of 0.9812 ± 0.0090 and 0.9633 ± 0.0173, respectively. Regarding boundary localization, TransUNet achieved the lowest HD (1.8935 ± 0.8570 mm), followed by Residual U-Net (1.9389 ± 1.7501 mm) and U-Net (1.9920 ± 1.6301 mm). Overall, the three models exhibited comparable segmentation performance. Residual U-Net showed the highest overlap-based performance according to both DSC and IoU, while TransUNet demonstrated the most favorable boundary distance. This means that evaluation based solely on overlap metrics may not fully reflect contour accuracy or the clinical reliability of the derived biometric measurements. These results suggest that the performance of the evaluated architectures varies according to the specific segmentation metric considered.

**Figure 8:**
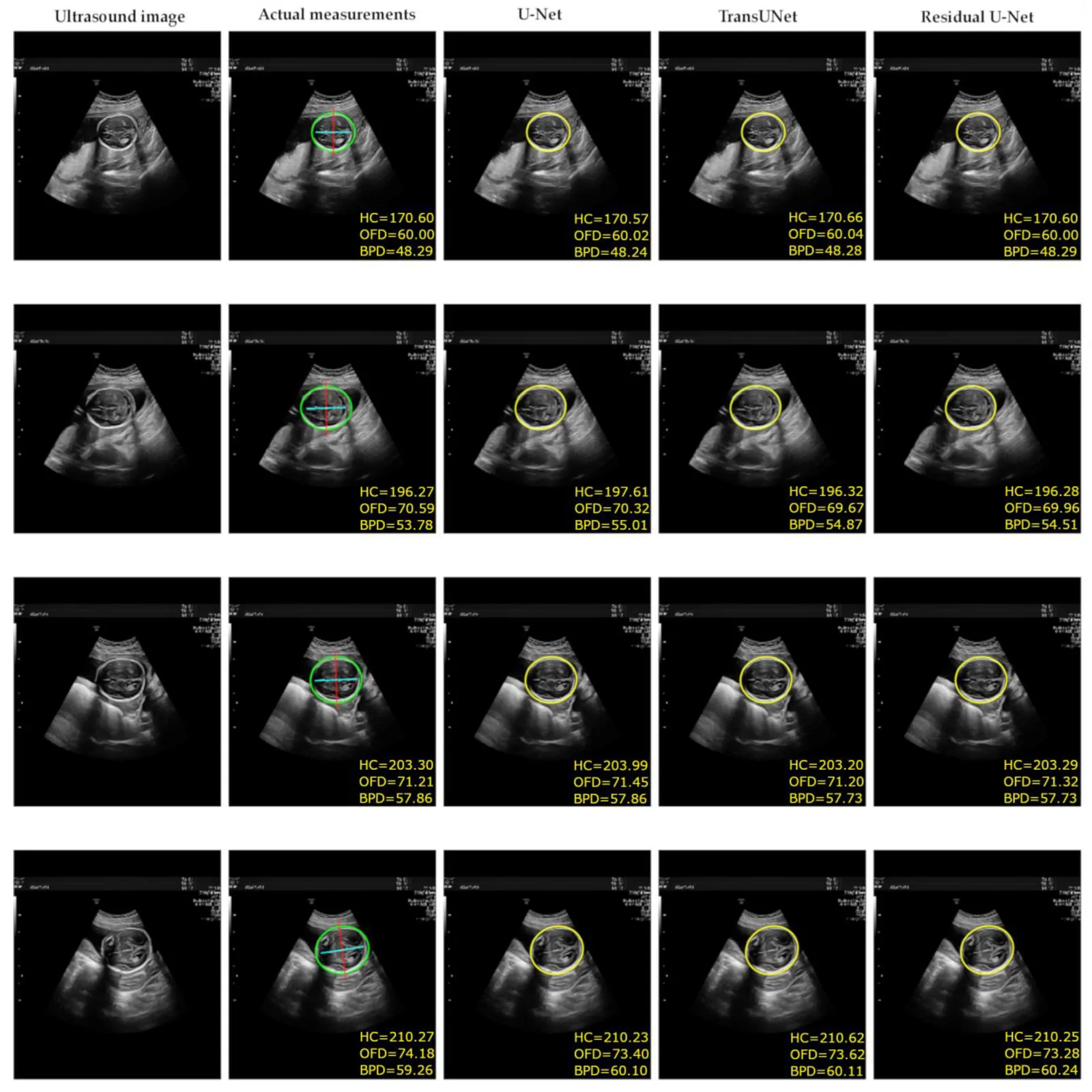
Representative fetal biometric measurements obtained from the predicted segmentation masks of the evaluated deep learning models (U-Net, Residual U-Net and TransUNet).

Figure 8 shows examples of automated fetal biometric measurements derived from the results of the evaluated deep learning models. Visual inspection reveals that all three models accurately reconstructed the fetal head contour. This enabled reliable ellipse fitting and subsequent estimation of HC, BPD and OFD. The estimated measurements closely matched the corresponding reference values in all cases examined. For instance, in the first case, all three models produced HC values that differed from the reference value by no more than 0.09 mm, whereas slight discrepancies were observed for BPD and OFD. Similar close agreement was observed in the remaining cases, although the models’ relative performance varied depending on the biometric parameter. In the second case, Residual U-Net produced an HC estimate of 196.28 mm compared to a reference value of 196.27 mm. U-Net, however, provided closer estimates for OFD (70.32 vs. 70.59 mm) and BPD (55.01 vs. 53.78 mm), which were slightly overestimated. These examples demonstrate that, even when the predicted contours appear visually similar, small geometric differences can result in measurable variations in the derived biometric parameters. Overall, the representative measurements are consistent with the quantitative findings, showing that Residual U-Net achieved the lowest MAE for HC and OFD, and TransUNet for BPD. Thus, these results provide qualitative evidence that the three architectures can generate clinically plausible biometric estimates while illustrating the parameter-specific differences in measurement accuracy observed in the quantitative analysis.

The biometric measurement results obtained on the test set are presented in Table 4 for HC, BPD and OFD. The MAE for HC ranged from 1.8877 to 1.9373 mm across the three models. The Residual U-Net model performed best, yielding an HC of 1.8877 ± 1.7689 mm. This was followed by the U-Net model (1.9280 ± 1.6549 mm) and the TransUNet model (1.9373 ± 1.7535 mm). For BPD, TransUNet produced the lowest MAE (0.7504 ± 0.7045 mm), followed by Residual U-Net (0.7713 ± 0.7206 mm) and U-Net (0.8027 ± 0.6571 mm). For OFD, Residual U-Net produced the lowest MAE (0.8672 ± 0.8342 mm). U-Net and TransUNet achieved MAE values of 0.8813 ± 0.7694 mm and 0.9054 ± 0.8442 mm, respectively. Overall, the MAE values were below 2 mm for HC, and below 1 mm for both BPD and OFD, across all evaluated models. These results demonstrate that the three architectures yielded relatively similar levels of biometric measurement error, although the model with the lowest error varied according to the specific biometric parameter. These results show that the proposed framework can produce reliable fetal biometric measurements from automatically segmented fetal head contours.

To increase the variability of the training data and improve the robustness of the model to variations in fetal head orientation and ultrasound appearance, data augmentation was applied exclusively to the training set. This process involved random horizontal flipping, rotation, scaling, brightness and contrast adjustment, and sliding transformations. These parameters were applied randomly during training. No augmentation was applied to the validation or the held-out test sets to ensure that model performance was evaluated on unmodified images. To further evaluate the impact of the adopted augmentation strategy, an additional experiment was conducted using the Residual U-Net, which exhibited the most robust biometric performance in the initial analysis. Using the augmented training set and keeping the test set unchanged, the model achieved Dice and IoU scores of 0.9806 ± 0.0081 and 0.9621 ± 0.0156, respectively. The corresponding Hausdorff distance was 1.9844 ± 0.7417 mm. The mean absolute errors for the derived biometric measurements were 2.0309 ± 1.7797 mm for HC, 1.0571 ± 0.9022 mm for OFD and 0.7985 ± 0.6497 mm for BPD. These results demonstrate that the proposed augmentation strategy maintains high segmentation performance while providing accurate biometric estimations on the held-out test set.

## 4. Discussion

This study evaluated an end-to-end approach for fetal head segmentation and biometric measurement calculation on a local clinical dataset. The results show that all three architectures such as U-Net, Residual U-Net and TransUNet achieved high segmentation performance. However, the study’s key finding is that evaluating segmentation performance using pixel-based metrics alone does not reflect clinical performance, and the accuracy of biometric measurements also needs to be considered.

The fact that all models achieved a DSC score above 98% on the dataset shows that fetal head segmentation can be carried out with a high level of accuracy using the latest deep learning architectures. These results are consistent with previous studies reporting the success of U-Net-based approaches in fetal ultrasound segmentation [4,9,22,39]. However, despite the relatively small differences in DSC scores among the models, the variation in HC measurement errors shows that high regional overlap does not necessarily equate to clinical measurement accuracy. This finding supports previous research suggesting that evaluating segmentation success in fetal biometry studies using metrics such as DSC or IoU alone may be insufficient [4,9,21,40].

In the experimental analysis, the U-Net and Residual U-Net models produced HC measurement errors that were highly comparable, with Residual U-Net achieving the lowest HC MAE (1.8877 mm). TransUNet, meanwhile, did not demonstrate a clear advantage. For OFD, the Residual U-Net model also achieved the lowest MAE (0.8672 mm), whereas the TransUNet model yielded the lowest BPD MAE (0.7504 mm). These findings are consistent with recent studies reporting that CNN-based architectures remain highly competitive under controlled clinical conditions [41,42]. Therefore, architectural performance appears to depend on the dataset and model selection may vary depending on factors such as image quality, device diversity and data heterogeneity. The relationship between segmentation metrics and biometric measurements has also produced notable results. While the TransUNet model produced the lowest HD in the dataset, it did not produce the lowest HC or OFD measurement error. Similarly, while the Residual U-Net model achieved the highest DSC and IoU, it did not provide the lowest error consistently across all biometric measurements. In addition, TransUNet achieved the lowest BPD MAE. This suggests that boundary accuracy and biometric measurement accuracy are not directly related. In ellipse-based biometric calculation methods, the general geometric form of the contour may be more important than local errors at specific boundary points [43]. Therefore, it has been concluded that model evaluation in clinical applications cannot be based solely on pixel overlap or boundary accuracy, and it also needs to directly incorporate clinically relevant measurement errors.

The Grad-CAM analysis demonstrated that all the models focused primarily on the fetal skull region when generating segmentation predictions. This indicates that the learned representations are anatomically meaningful. However, the different attention distributions observed among the models suggest that comparable segmentation performance can be achieved using different feature extraction strategies. This may partly explain the differences observed in biometric measurement accuracy. Grad-CAM was used to create a visual representation of the image regions that contributed to the segmentation output of the evaluated architectures. As Grad-CAM requires a convolutional feature representation and a scalar target, the segmentation output was used to define the target for gradient computation. The gradients of this target with respect to the feature maps of the selected convolutional layer were then calculated. The resulting channel-wise weighted activation maps were aggregated to generate the Grad-CAM heatmap. These heatmaps were then normalized and overlaid on the corresponding ultrasound images. The target layer was selected based on the internal architecture of each model rather than using an identical layer name across all networks. For U-Net and Residual U-Net, the convolutional layer in the first decoder up-sampling block, directly after the bottleneck, was chosen. These layers occupy the same functional and structural position in both architectures, enabling consistent comparison of the two CNN-based models. For TransUNet, however, the corresponding layer was selected from the decoder submodule due to the architecture’s different internal organization. Therefore, despite the exact layer location differing between architectures, the selected layers still represent comparable decoder-level feature representations that are used to reconstruct the segmentation output.

**Figure 9:**
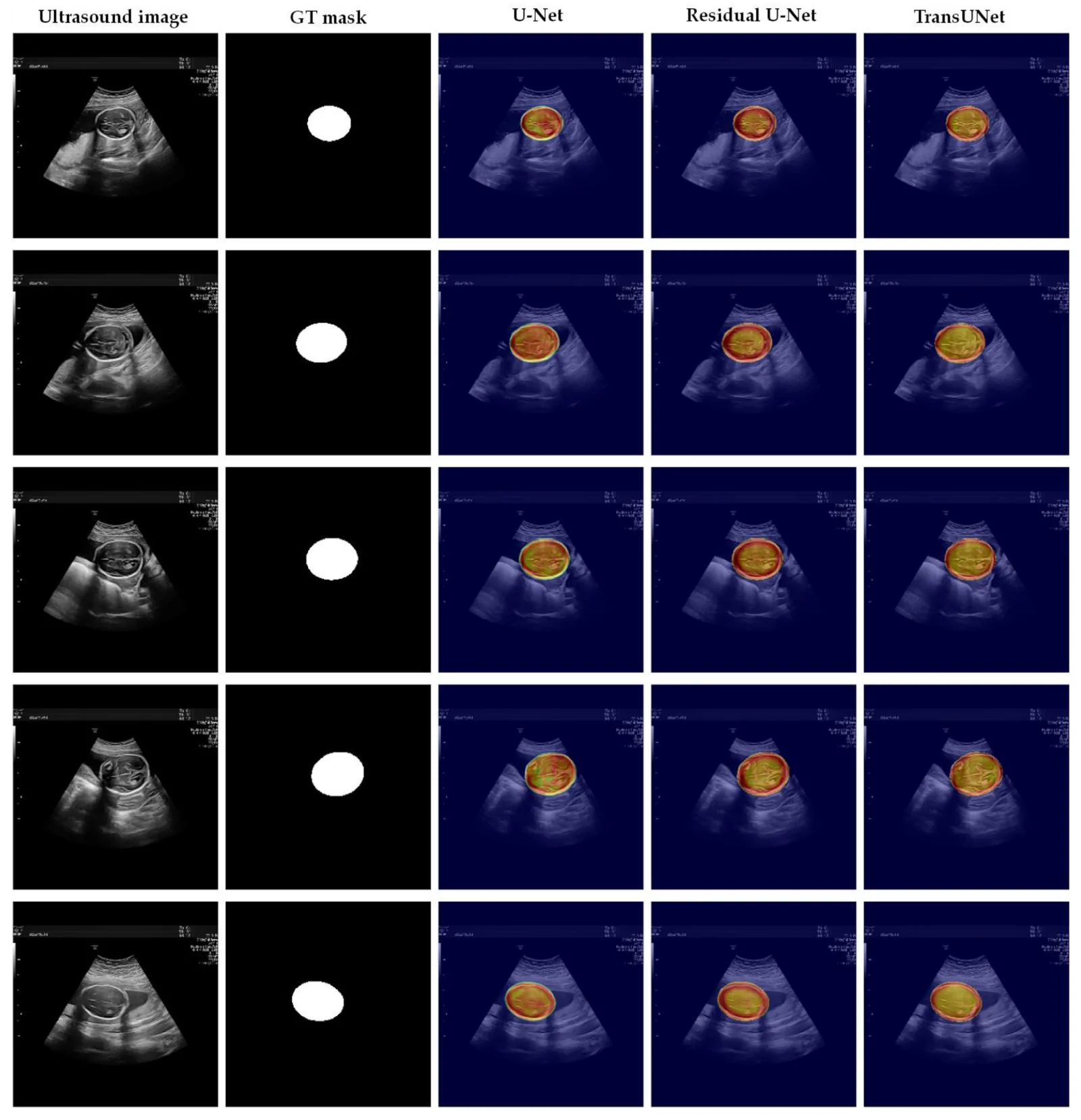
Grad-CAM visualizations of the evaluated deep learning models for fetal head segmentation.

Figure 9 shows representative Grad-CAM visualizations of the three models that were evaluated for segmentation. The highest activation responses are consistently concentrated within and around the fetal head region across the examples, while relatively low activation is observed in the surrounding background and non-target regions. This suggests that all three models primarily use image features from the anatomically relevant region for segmentation. Notably, the Residual U-Net model generally exhibited more continuous and spatially coherent activation patterns across the fetal head and its boundary, suggesting a more consistent representation of the target anatomy. In contrast to others, Residual U-Net produced broader, more continuous activations that covered the fetal head region. This pattern is consistent with the residual feature-learning mechanism, which may facilitate the preservation and integration of anatomical features across network depths. The activation pattern of Residual U-Net is also qualitatively consistent with its strong biometric performance, as evidenced by the relatively low measurement errors. TransUNet concentrated its activations on the fetal head as well, but also exhibited somewhat broader activation regions. This is consistent with its ability to incorporate contextual information through Transformer-based feature modelling. Nevertheless, these Grad-CAM visualizations can be used to provide qualitative evidence of spatial feature activation, but they should not be considered as definitive proof of explainability, reliability or the causal use of specific anatomical features. In particular, the similarity of the activation maps across models does not demonstrate a direct correlation between the observed attention patterns and the slight variations in biometric errors. Therefore, Grad-CAM is considered to complement the quantitative segmentation and biometric analyses. Overall, the visualizations demonstrate that the Residual U-Net model focuses coherently on the fetal head region, which supports its strong performance in measurement-oriented evaluations. However, formal validation of explainability would require additional dedicated analyses.

To determine whether the differences between the three architectures (U-Net, Residual U-Net and TransUNet) were statistically significant, a two-sided Wilcoxon signed-rank test was performed on the same 288 test samples to make pairwise comparisons. The paired design was used because each model was used to generate predictions for the same set of test images. Statistical significance was assessed at a significance level of *p < 0.05*. The analysis was performed separately for DSC, absolute HC error, absolute OFD error and absolute BPD error. A statistically significant difference was observed for DSC between U-Net and Residual U-Net (*p = 0.0172*), and also between Residual U-Net and TransUNet (*p = 0.0119*). However, no significant difference was revealed between Basic U-Net and TransUNet (*p = 0.4726*). None of the pairwise comparisons for HC absolute error reached statistical significance. Similarly, no statistically significant differences were observed for OFD absolute error in the three respective pairwise comparisons. However, a statistically significant difference was observed for BPD absolute error between Basic U-Net and TransUNet (*p = 0.0385*). In contrast, the comparisons between U-Net and Residual U-Net (*p = 0.1702*) and between Residual U-Net and TransUNet (*p = 0.5814*) were not statistically significant. Overall, the pairwise analysis showed that statistically significant differences were primarily observed in segmentation overlap, whereas biometric measurement errors were largely comparable across the three architectures.

To investigate whether GA influences the biometric performance of the proposed framework, an additional subgroup analysis was performed on the held-out test set. The 288 test images were divided into two groups according to GA: 15–20 weeks (n = 90) and 21–24 weeks (n = 198). The Residual U-Net, which had previously been trained and demonstrated the lowest overall MAE in HC among the evaluated architectures, was used without any additional training or fine-tuning. Therefore, the subgroup analysis was performed exclusively on the held-out test samples and did not involve retraining or modification of the model. The absolute HC measurement errors obtained for the two GA groups were compared using a two-sided Mann–Whitney U test. The mean absolute HC measurement error using the previously trained Residual U-Net was 1.8212 ± 1.8928 mm in the 15-20 week group and 1.9179 ± 1.7138 mm in the 21–24 week group. According to the Mann–Whitney U test, the difference between the two groups was not statistically significant (*p = 0.2808*). These results imply that HC measurement performance did not demonstrate a statistically significant dependence on GA range represented in the dataset.

The significance of the observed biometric errors is also important when considered alongside the variability of routine fetal ultrasound measurements. Previous studies have demonstrated that fetal biometric measurements are prone to interobserver variability, even among trained sonographers. Sarris et al. [13] observed 95% limits of agreement for HC between observers, emphasizing the inherent variability associated with routine ultrasound biometry. Similarly, another study of the reproducibility of fetal head measurements showed interobserver differences of less than 1% on average, with 95% limits of agreement for BPD, OFD and HC measurements [8]. By way of comparison, this study demonstrated relatively low absolute measurement errors, with the lowest MAE values of 1.8877 mm for HC, 0.7504 mm for BPD, and 0.8672 mm for OFD across the evaluated models. These results suggest that the proposed measurement-oriented framework can achieve submillimeter mean absolute errors for BPD and OFD, with errors in HC estimation remaining below 2 mm. However, these findings should be interpreted with caution in relation to the inter-observer variability reported in clinical practice, since this study did not include independent biometric measurements performed by multiple experts. Consequently, the reported MAE values primarily reflect agreement between automated and available reference measurements, rather than providing direct evidence of equivalence to expert-level inter-observer variability.

The reliability of automated fetal biometry also depends on the quality and consistent standardization of the ultrasound plane acquired. Current International Society of Ultrasound in Obstetrics and Gynecology (ISUOG) recommendations emphasize the use of a standardized transthalamic plane for fetal head biometry, including appropriate visualization. In routine practice, however, images may differ from the ideal transthalamic plane due to fetal position, maternal factors, limited acoustic windows or differences in image acquisition caused by the operator. These deviations can alter the apparent cranial geometry, which can affect the fitted ellipse and the derived HC, BPD and OFD measurements, even when the fetal head has been successfully segmented. In particular, acquiring images may introduce systematic changes to the apparent biparietal and occipitofrontal dimensions, affecting the reliability of ellipse-based estimation of HC. Therefore, the high measurement accuracy observed in this study needs to be interpreted in the context of images selected to represent the standard fetal head measurement plane. For routine clinical use, an automated plane-quality assessment tool may be necessary to identify non-standard acquisitions before biometric measurements are generated.

Several limitations need to be acknowledged. A major limitation of this study is that the dataset comprised only healthy singleton pregnancies. While this design provided a relatively consistent population with which to evaluate the proposed segmentation and measurement framework, it limits the generalizability of the findings to the wider field of fetal medicine. In particular, the current results may not apply directly to pregnancies complicated by fetal growth restriction, microcephaly, macrocephaly, ventriculomegaly, cranial malformations or other abnormalities that could affect fetal cranial morphology. In addition, pathological changes in skull shape, size, contour or anatomical proportions may affect both the segmentation process and subsequent ellipse-based biometric measurements. Secondly, the proposed framework was developed using data acquired from a single clinical center with a single ultrasound platform (GE Voluson E8), and external validation using independently acquired data was not performed. While the dataset reflects the variability encountered in routine clinical practice, multicenter validation using images acquired with different ultrasound systems would strengthen its generalizability further. Therefore, the results cannot be interpreted as evidence of immediate clinical readiness or applicability to a wide range of institutions and imaging systems. Thirdly, the imaging conditions encountered in routine fetal examinations may also be substantially more challenging. Factors such as maternal obesity, oligohydramnios, an unfavorable fetal position, reduced acoustic windows and other technically challenging examinations can lead to increased image noise, attenuation, reduced contrast, shadowing and incomplete visualization of the standard fetal head plane. These factors may further compromise the accuracy of automated segmentation and biometric measurements. Fourthly, an important methodological consideration is converting image-based measurements into physical units. To avoid inaccuracies associated with a fixed pixel-to-millimeter conversion factor, the base physical scale was derived from the depth reference of the ultrasound device and adjusted according to the resizing factor specific to each image. This approach was appropriate for the present dataset, as all images were acquired using the same ultrasound system, resolution and imaging depth. However, external validation is required for images obtained using different ultrasound systems or acquisition settings. Fifthly, another limitation of this study is that image-level data were used for splitting rather than pregnancy-level data. Although exact duplicates were removed using pixel-level MD5 hashing, images from the same pregnancy could still have been distributed across different subsets. This could potentially introduce data dependency and optimistic performance estimates. Therefore, the reported results need to be seen primarily as a controlled comparison of the evaluated architectures, rather than as an independent estimate of generalizability at the patient level. Future studies require larger datasets with pregnancy-level splitting. Lastly, only three segmentation architectures were evaluated in this study. Future studies may investigate recently developed models and advanced medical vision transformers to further explore the relationship between segmentation quality and biometric accuracy. Furthermore, future studies are needed to validate the proposed framework using larger, multicenter, clinically diverse sample groups that include both normal and pathological pregnancies. These studies could also examine whether disease-specific adaptation, targeted augmentation or domain adaptation strategies are necessary to ensure consistent segmentation and biometric accuracy across various clinical conditions. Additionally, the proposed study focused exclusively on fetal head biometry. Extending the measurement-oriented evaluation framework to other fetal biometric parameters, such as abdominal circumference and femur length, would validate its clinical applicability further.

## 5. Conclusions

This study presented a deep learning framework oriented towards measurements for the automated biometry of the fetal head from prenatal ultrasound images. This framework integrates fetal head segmentation with the extraction of biometric measurements based on ellipses. Three deep learning architectures—U-Net, Residual U-Net and TransUNet—were evaluated using a locally acquired clinical dataset under identical conditions. Unlike previous studies, which focused primarily on overlap-based segmentation metrics, the proposed framework assessed both segmentation quality and the clinical accuracy of the derived biometric measurements simultaneously.

The experimental results showed that all the models achieved excellent segmentation performance, with DSC values above 0.98 and IoU values exceeding 0.96. However, a comparative analysis revealed that no single model outperformed the others consistently across all evaluation criteria. Residual U-Net produced the highest overlap-based segmentation accuracy, with the highest DSC and IoU values, while TransUNet produced the lowest HD. In terms of biometric measurements, Residual U-Net yielded the lowest MAE for HC and OFD, whereas TransUNet achieved the lowest MAE for BPD. These results demonstrate that conventional segmentation metrics alone are insufficient for identifying the best model for automated fetal head analysis.

A key finding of this study is that a clinically meaningful evaluation needs to consider more than just segmentation overlap. It also needs to assess the accuracy of biometric measurements directly. The results suggest that preserving contour geometry and global anatomical shape is more important for reliable fetal biometry than maximizing pixel-wise overlap alone. Consequently, a measurement-oriented evaluation provides a more comprehensive and clinically relevant framework for assessing deep learning models in obstetric ultrasound. The proposed framework benefits from using a locally acquired clinical dataset representing routine obstetric practice, as well as integrating explainability analysis through Grad-CAM. This improves the transparency and clinical interpretability of the developed models.

Conclusively, the findings demonstrate the technical feasibility of combining deep learning-based fetal head segmentation with automated biometric measurement extraction, while also highlighting the value of a measurement-oriented evaluation strategy. However, as this study was conducted at a single institution using a single ultrasound platform and a cohort of healthy singleton pregnancies, it does not establish clinical effectiveness, generalizability or readiness for routine clinical implementation. Before widespread clinical adoption can be considered, prospective multicenter external validation involving diverse patient populations, ultrasound systems, acquisition protocols and operator experience levels, together with direct comparison against experts, is necessary. Future studies could also investigate the framework’s performance in pathological and technically challenging pregnancies, as well as evaluate additional fetal biometric parameters. Furthermore, future studies could build on the proposed measurement-oriented framework by extending it to include more complex ultrasound biomarkers, such as fetal and placental vascular parameters derived from color Doppler imaging, in addition to conventional fetal biometry.

## Figures and Tables

**Table 1:** Characteristics of the fetal ultrasound dataset used in this study.

Characteristic	Value
Institution	Bursa City Hospital, Bursa, Türkiye
Study period	July 2025–January 2026
Study design	Retrospective
Number of pregnancies	206
Pregnancy type	Healthy singleton pregnancies
Maternal age	18-40 years
Gestational age	15-24 weeks
Ultrasound system	GE Voluson E8
Transducer	C2-9 MHz convex probe
Data source	Prenatal ultrasound videos
Final dataset size	1918 image-mask pairs
Annotation type	Pixel-wise fetal head masks
Annotation reviewer	A radiologist with experience, working in collaboration with a specialist obstetrician
Pixel calibration	140 mm/549 pixels = 0.2550 mm/pixel
Data split	70% training, 15% validation, 15% test
Random seed	42
Ethics approval	Bursa City Hospital Ethics Committee (2026-06/3)
Informed consent	Obtained from all participants

**Table 2:** The basic training configurations with hyperparameters and model implementation for deep learning models in proposed study.

Parameter	Value
Framework/Language	PyTorch/Python 3.10
GPU	NVIDIA GeForce GTX 1080 (8 GB VRAM)
Optimizer	Adam (CNN-based models)/SGD with momentum (TransUNet)
Initial learning rate	1 × 10^−4^ (CNN-based models)/1 × 10^−2^ (TransUNet)
Learning rate scheduler	ReduceLROnPlateau (CNN-based models)/Polynomial decay (TransUNet)
Loss function	Combined BCE and Dice loss
Batch size	2-4 (CNN-based models)/1–2 (TransUNet)
Number of epochs	50
Gradient clipping	Maximum norm = 1.0
Mixed-precision instruction	Enabled Active (AMP)
Random seed	42
Trainable parameters	7.765.409 (U-Net)/8.115.073 (Residual U-Net)/105.322.001 (TransUNet)

**Table 3:** Segmentation performance of U-Net, Residual U-Net, and TransUNet on the test set.

Architecture	IoU	DSC	HD [mm]
U-Net	0.9633 ± 0.0173	0.9812 ± 0.0090	1.9920 ± 1.6301
TransUNet	0.9638 ± 0.0090	0.9815 ± 0.0101	1.8935 ± 0.8570
Residual U-Net	0.9647 ± 0.0196	0.9819 ± 0.0102	1.9389 ± 1.7501

**Table 4:** Comparison of biometric measurement accuracy obtained by the evaluated deep learning models.

Architecture	HC [MAE, mm]	BPD [MAE, mm]	OFD [MAE, mm]
U-Net	1.9280 ± 1.6549	0.8027 ± 0.6571	0.8813 ± 0.7694
TransUNet	1.9373 ± 1.7535	0.7504 ± 0.7045	0.9054 ± 0.8442
Residual U-Net	1.8877 ± 1.7689	0.7713 ± 0.7206	0.8672 ± 0.8342

## Data Availability

The data presented in this study are available on request from the corresponding author due to ethical restrictions.
